# Development and validation of a nomogram to predict the five-year risk of revascularization for non-culprit lesion progression in STEMI patients after primary PCI

**DOI:** 10.3389/fcvm.2023.1275710

**Published:** 2023-11-29

**Authors:** Feng Dai, Xianzhi Xu, Chunxue Zhou, Cheng Li, Zhaoxuan Tian, Zhaokai Wang, Shuping Yang, Gege Liao, Xiangxiang Shi, Lili Wang, Dongye Li, Xiancun Hou, Junhong Chen, Tongda Xu

**Affiliations:** ^1^ Department of Cardiology, The Affiliated Hospital of Xuzhou Medical University, Xuzhou, China; ^2^ Department of Oral Medicine, School of Stomatology, Xuzhou Medical University, Xuzhou, China; ^3^ Department of General Practice & Geriatrics, The Affiliated Hospital of Xuzhou Medical University, Xuzhou, China; ^4^ Department of Nuclear Medicine, The Affiliated Hospital of Xuzhou Medical University, Xuzhou, China

**Keywords:** non-culprit lesion, percutaneous coronary intervention, nomogram, ST-segment elevation myocardial infarction, primary PCI

## Abstract

**Background:**

Acute ST-segment elevation myocardial infarction (STEMI) patients after primary PCI were readmitted for revascularization due to non-culprit lesion (NCL) progression.

**Objective:**

To develop and validate a nomogram that can accurately predict the likelihood of NCL progression revascularization in STEMI patients following primary PCI.

**Methods:**

The study enrolled 1,612 STEMI patients after primary PCI in our hospital from June 2009 to June 2018. Patients were randomly divided into training and validation sets in a 7:3 ratio. The independent risk factors were determined by LASSO regression and multivariable logistic regression analysis. Multivariate logistic regression analysis was utilized to develop a nomogram, which was then evaluated for its performance using the concordance statistics, calibration plots, and decision curve analysis (DCA).

**Results:**

The nomogram was composed of five predictors, including age (OR: 1.007 95% CI: 1.005–1.009, *P < *0.001), body mass index (OR: 1.476, 95% CI: 1.363–1.600, *P < *0.001), triglyceride and glucose index (OR: 1.050, 95% CI: 1.022–1.079, *P < *0.001), Killip classification (OR: 1.594, 95% CI: 1.140–2.229, *P *= 0.006), and serum creatinine (OR: 1.007, 95% CI: 1.005–1.009, *P < *0.001). Both the training and validation groups accurately predicted the occurrence of NCL progression revascularization (The area under the receiver operating characteristic curve values, 0.901 and 0.857). The calibration plots indicated an excellent agreement between prediction and observation in both sets. Furthermore, the DCA demonstrated that the model exhibited clinical efficacy.

**Conclusion:**

A convenient and accurate nomogram was developed and validated for predicting the occurrence of NCL progression revascularization in STEMI patients after primary PCI.

## Introduction

Coronary artery disease causes 8.9 million patient deaths and 264 million workforce losses annually (1). Among the various manifestations of coronary artery disease, ST-elevation myocardial infarction (STEMI) is the most severe type characterized by rapid onset, progression, and high morbidity and mortality (2). Fortunately, the availability of thrombolytic therapy and primary percutaneous coronary intervention (PCI) has significantly improved STEMI patients' prognosis. Although the utilization of drug-eluting stents has contributed to reducing the occurrences of in-stent restenosis (3). Revascularization after PCI remains a common clinical problem. The rate of revascularization after PCI was 12% at 1 year, 15% at 2 years, 20% at 4 years, and 32.3% at 5 years (4, 5). The in-stent restenosis is not the sole cause of repeat revascularization. One study highlighted that non-culprit lesion (NCL) progression accounts for over half of all revascularizations (4). NCL progression refers to developing or worsening atherosclerotic lesions outside the culprit lesions that cause ischemia, angina, and myocardial infarction. Such progression can lead to recurrent ischemia, angina, myocardial infarction, or death. Nonetheless, not all non-culprit lesions necessitate revascularization. Hence, predicting the likelihood of revascularization for NCL progression in STEMI patients is crucial.

Several studies have examined risk factors associated with repeat revascularization, such as fasting glucose, peak cTnI levels, and complex lesions (6, 7). Compared to conventional statistical models, the nomogram provides several advantages such as validation of effectiveness, visual representation, and personalized risk assessment (8). The nomogram effectively quantifies risk factors and synthesizes them into a predictive score to inform clinical decisions.

However, there are no studies for developing and validating a nomogram specifically for NCL progression revascularization. The study aims to build and validate a nomogram to predict the likelihood of NCL progression revascularization in STEMI patients after complete revascularization.

In this retrospective study, a nomogram was established to screen high-risk groups based on our clinical results, which may provide guidance for clinicians and assesses STEMI patients' prognosis.

### Study population

The study was conducted in accordance with the Declaration of Helsinki and approved by the Ethics Committee of the Affiliated Hospital of Xuzhou Medical University (XYFY2022-KL375-01). Since the study was retrospective, the committee waived the requirement for written informed consent. Personal private information was removed prior to the data is analyzed.

7,277 patients who underwent primary PCI for STEMI between 2009 and 2018 were initially screened in the affiliated hospital of Xuzhou medical university. Patients showing multivessel disease at the first CAG were included in the study only if NCL with diameter stenosis greater than 70% were treated. Secondly, 2,469 patients combined of at least one NCL (diameter stenosis between 50% and 70%) were readmitted to the hospital within 5 years after primary PCI for clinical symptoms such as chest pain and chest tightness. These patients underwent review coronary angiography (CAG). Finally, 1,612 patients were included in the study according to the exclusion and inclusion criteria.

Inclusion criteria: (1) diagnosis of acute ST-segment elevation myocardial infarction (9), (2) successful drug-eluting stent implantation procedure, (3) immediate complete revascularization or elective completion of complete revascularization within 2 months (10), (4) no in-hospital cardiovascular events such as cardiogenic shock, recurrent myocardial infarction, or death, (5) follow-up CAG within 5 years after the first PCI, (6) no anticoagulant or antiplatelet contraindications, (7) over 18 years old, (8). Combination of at least one NCL (diameter stenosis between 50% and 70%).

Exclusion criteria: (1) incomplete clinical data (*n*=576), (2) follow-up CAG showing in-stent restenosis or recommendation for coronary artery bypass graft (*n*=258), (3) severe hepatic insufficiency or renal insufficiency (*n*=23).

### First PCI procedure

Patients undergoing the procedure were routinely administered stress dose medications (300 mg of aspirin, 600 mg of clopidogrel, or 180 mg of ticagrelor). To ensure proper anticoagulation, 3,000 IU of unfractionated heparin and 200 ug of nitroglycerin were administered prior to CAG. The administration of heparin was initiated at 100 IU/kg before the operation, with the option of adding 1,000 IU/h during the procedure. Additionally, intraoperative medications such as nitroprusside, nitroglycerin, tirofiban, and other drugs aimed at improving coronary ischemia were selectively administered based on the patient's condition.

Following the operation, medications were prescribed according to established guidelines. These included: (1) a dual antiplatelet regimen involving aspirin (100 mg once daily) combined with either clopidogrel (75 mg once daily) or ticagrelor (90 mg twice daily); (2) statin; (3) angiotensin converting enzyme inhibitor or angiotensin receptor blocker; and (4) beta-blocker.

Senior cardiologists performed both CAG and PCI procedures. Two skilled clinicians thoroughly analyzed all CAG images.

### Clinical endpoints and definitions

The clinical endpoint was that STEMI patient readmitted with ischemic symptoms found to have NCL progression (diameter stenosis progresses from between 50%–70% to >70%) requiring revascularization within 5 years after PCI. NCL was defined as a vessel with stenosis between 50% and 70% at the first CAG (7). All patients underwent primary PCI of the culprit lesions successfully. Patients showing multivessel disease at the first CAG were included in the study only if they had completed complete revascularization or selective complete revascularization. Complete revascularization was defined as successful revascularization of all coronary artery lesions or segments ≥1.5 mm in diameter with ≥70% diameter stenosis regardless of their functional significance (11). Related definitions included the following: Calcified lesions were defined as the presence of moderate or severe calcification in the vessel wall (12); bifurcation lesions were defined as stenosis adjacent to and/or involving the opening of a major branch (13); ostial lesions were defined as lesions within 3 mm of the origin of the major coronary arteries (14); angular distortion lesions were defined as angles of at least one major branch of the coronary artery ≥45° along the direction of the main coronary artery (15).

### Data collection

All patient clinical features (including demographics, previous history, laboratory indices, two-times coronary angiography images, and medication use at discharge) were collected. Each patient's blood was collected within 24 h of admission, and the central laboratory tested all laboratory parameters before operation. Data collection during PCI consisted of door-to-balloon time, calcified lesions, bifurcation lesions, open and angular distortion lesions, and culprit lesions. Echocardiography measures the left ventricular ejection fraction.

### Data analysis

Categorical variables were displayed as counts and percentages and then compared using either the *χ*^2^ or Fisher exact test. Meanwhile, continuous variables were presented as mean ± standard deviation. If the variables adhered to a normal distribution pattern, they were compared using the *t*-test. However, if the variables exhibited non-normal distribution, they were compared using the Mann–Whitney *U*-test. Univariate logistic regression was analyzed to filter significant variables in the training cohort. This part was done using SPSS version 25.0 (SPSS Inc., Chicago, IL, USA). LASSO regression was used to screen out non-zero coefficient characteristics, and multivariate logistic regression backward stepwise regression was used to analyze the independent predictors. The restricted cubic spline was performed to investigate the linear relationships between continuous variables and NCL progression revascularization. A nomogram was developed using variables with *P* < 0.05 based on the result of the multivariate logistic regression. The discrimination capability of the model was gauged by the concordance index, which is equal to the area under the receiver operating characteristics curve (AUC-ROC). This metric was used to assess the effectiveness of the nomogram's discrimination capacity. In order to determine the level of accuracy of the calibration, Hosmer-Lemeshow tests and calibration plots were carried out. Clinical efficacy was evaluated using decision curve analysis. The statistical threshold for determining significance was established at a level of *P* < 0.05 for the two-sided test. This part was analyzed using R Studio version 4.1.3 (https://cran.r-project.org).

## Results

**Figure 1:**
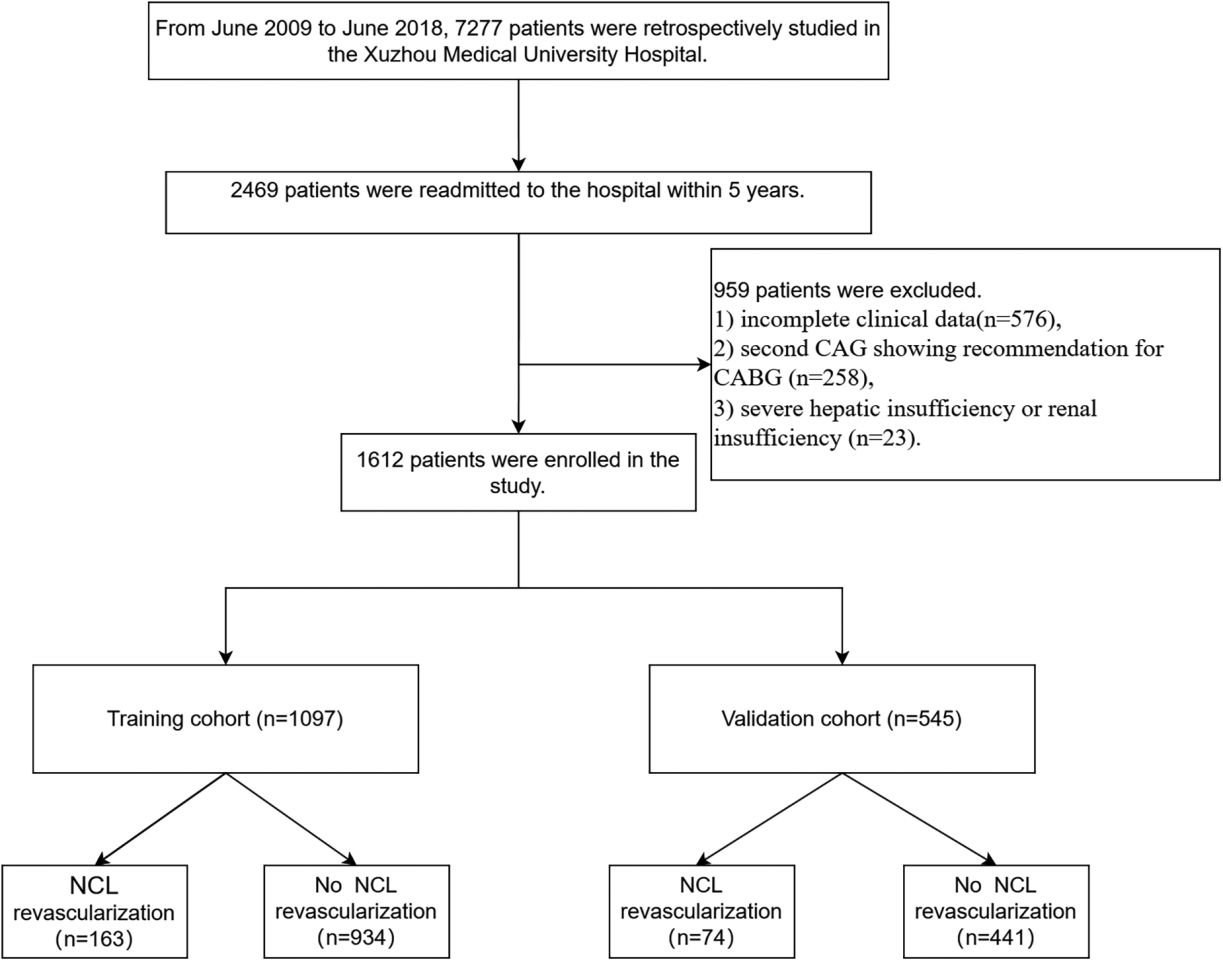
The study flowchart for developing and validating nomograms. CAG, coronary angiography; CABG, coronary artery bypass graft.

**Figure 2:**
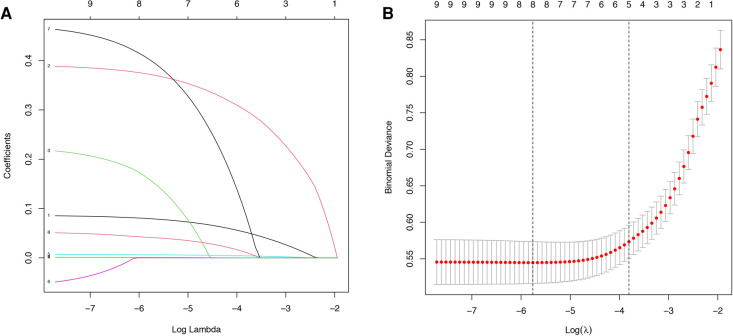
Variable screening based on lasso regression. (**A**) Characterization of the variation of variable coefficients; (**B**) The process of selecting the optimal value of the parameter *λ* in the Lasso regression model by the cross-validation method.

**Figure 3:**
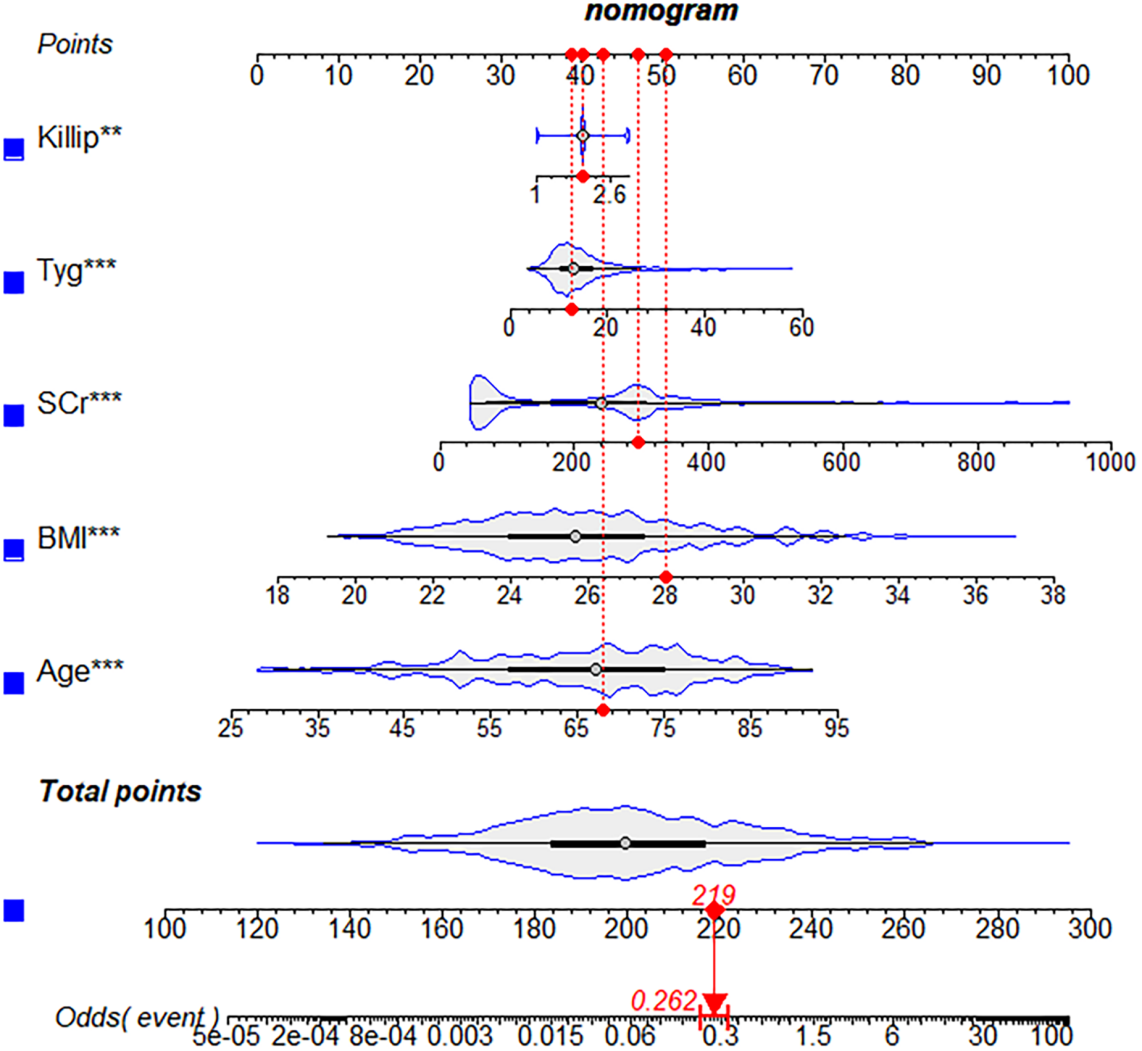
A nomogram for predicting the possibility of revascularization for non-culprit lesions progression. BMI, body mass index; SCr, serum creatinine.

The study flow chart is shown in Figure 1. There are 1,612 STEMI patients were enrolled based on inclusion and exclusion criteria. Patients were randomly divided into training and validation sets in a 7:3 ratio. Among them, 14.8% (163/1,097) of patients in the training set underwent repeat PCI due to NCL progression, similar to 14.3% (74/515) of patients in the validation set. Table 1 presents a comprehensive sight of the baseline characteristics of the two sets. Table 2 displays the results of the univariate logistic regression. Univariate logistic regression analysis showed that the following factors were consistently associated with NCL revascularization in STEMI patients following primary PCI: age (OR: 1.09, 95% CI: 1.07–1.11, *P *< 0.001), body mass index (BMI) (OR: 1.55, 95% CI: 1.44–1.67, *P *< 0.001), triglyceride and glucose index (OR: 1.03, 95% CI: 1.01–1.05, *P* = 0.005), Killip classification (OR: 1.76, 95% CI: 1.31–2.35, *P *< 0.001), Hypertension (OR: 0.62, 95% CI: 0.44–0.86, *P* = 0.005), serum creatinine (SCr) (OR: 1.01, 95% CI: 1.00–1.01, *P *< 0.001), Lipoprotein-a (OR: 1.00, 95% CI: 1.00–1.00, *P *< 0.001), creatine kinase MB (OR: 1.00, 95% CI: 1.00–1.01, *P* = 0.001), total cholesterol (OR: 2.59, 95% CI: 2.18–3.08, *P *< 0.001). LASSO regression analysis showed that age, BMI, triglyceride and glucose index, Killip classification, and SCr were the more important predictors with non-zero coefficient, as shown in Figure 2. Multivariate logistic regression analysis identified in Table 3 that age (OR: 1.007, 95% CI: 1.005–1.009, *P *< 0.001), BMI (OR: 1.476, 95% CI: 1.363–1.600, *P *< 0.001), triglyceride and glucose index (OR: 1.050, 95% CI: 1.022–1.079, *P *< 0.001), Killip classification (OR: 1.594, 95% CI: 1.140–2.229, *P* = 0.006), and SCr (OR: 1.007, 95% CI: 1.005–1.009, *P *< 0.001). A nomogram was built and displayed in Figure 3. A point value is projected at the top of the nomogram for each independent predictor. This process helps obtain a score ranging between 0 and 100. The scores of all variables are added to get a total score, which is projected down to a vertical line on the “Risk” line, indicating the risk of NCL revascularization. A higher overall score indicates a higher likelihood of NCL revascularization. Consequently, the model could visually predict the occurrence of NCL progression revascularization.

**Table 1:** Participant characteristics.

Variable	Cohort, No. (%)		*P*-value
	Training set, *N* = 1,097	Validation set, *N* = 515	
Intervals between coronary angiograms, months	41 (34, 47)	42 (35, 48)	0.342
Age, years	67 (57, 75)	66 (56, 75)	0.41
Sex, *n* (%)			0.968
Male	802 (73%)	377 (73%)	
Female	295 (27%)	138 (27%)	
Body mass index, kg/m2	25.64 (23.96, 27.42)	25.33 (23.80, 27.10)	0.057
Diabetes mellitus, *n* (%)			0.093
Yes	829 (76%)	369 (72%)	
No	268 (24%)	146 (28%)	
Hypertension, *n* (%)			0.361
Yes	555 (51%)	248 (48%)	
No	542 (49%)	267 (52%)	
Smoking status, *n* (%)			0.459
Yes	458 (42%)	205 (40%)	
No	639 (58%)	310 (60%)	
Alcohol drinking status, *n* (%)			0.954
Yes	146 (13%)	68 (13%)	
No	951 (87%)	447 (87%)	
Coronary heart disease, *n* (%)			0.184
Yes	34 (3%)	10 (2%)	
No	1,063 (97%)	505 (98%)	
Transient ischemic attack, *n* (%)			0.275
Yes	50 (5%)	30 (6%)	
No	1,047 (95%)	485 (94%)	
Aspirin, *n* (%)			1
Yes	1,097 (100%)	515 (100%)	
No	0 (0%)	0 (0%)	
Clopidogrel, *n* (%)			0.290
Yes	404 (37%)	175 (34%)	
No	693 (63%)	340 (66%)	
Ticagrelor, *n* (%)			0.510
Yes	671 (61%)	324 (63%)	
No	426 (39%)	191 (37%)	
Beta-blockers, *n* (%)			0.75
Yes	883 (80%)	418 (81%)	
No	214 (20%)	97 (19%)	
ACEI/ARB, *n* (%)			0.224
Yes	623 (57%)	309 (60%)	
No	474 (43%)	206 (40%)	
Calcium channel blockers, *n* (%)			0.467
Yes	139 (13%)	72 (14%)	
No	958 (87%)	443 (86%)	
Statins, *n* (%)			0.102
Yes	1,080 (98%)	512 (99%)	
No	17 (2%)	3 (1%)	
High intensity of statin, *n* (%)			0.657
Yes	401 (37%)	182 (35%)	
No	696 (63%)	333 (65%)	
Door-to-balloon time, min	295.24 ± 43.67	299.07 ± 69.13	0.177
Left ventricular ejection fraction, %	56 (50, 61)	55 (51, 60)	0.062
Creatine kinase MB, ng/ml	22 (5, 60)	24 (4, 97)	0.359
White blood cell, ×109/L	9.10 (7.40, 11.10)	9.10 (7.00, 11.30)	0.351
Neutrophile, ×10^9^/L	6.81 (5.18, 8.72)	6.80 (6.34, 7.40)	0.236
Lymphocyte, ×10^9^/L	1.50 (1.00, 2.00)	1.40 (1.00, 2.00)	0.172
High-sensitive C-reactive protein, mg/L	8 (3, 26)	5 (2, 31)	0.459
Fasting plasma glucose, mmol/L	5.5 (4.1, 7.4)	5.6 (3.6, 8.4)	0.998
Serum creatinine, umol/L	239 (69, 305)	228 (152, 287)	0.517
Serum uric acid, umol/L	308 (258, 355)	307 (302, 311)	0.656
Total cholesterol, mmol/L	1,007 (809, 1,169)	939 (544, 1,748)	0.221
Triglyceride, mmol/L	4.30 (3.73, 5.02)	4.27 (3.68, 4.97)	0.311
Triglyceride and glucose index	1.36 (0.98, 1.92)	1.30 (0.90, 1.94)	0.136
High-density lipoprotein cholesterol, mmol/L	1.03 ± 0.36	1.02 ± 0.29	0.588
Low Density Lipoprotein cholesterol, mmol/L	2.73 ± 0.88	2.68 ± 0.85	0.192
Low Density Lipoprotein cholesterol on re-admission, mmol/L	2.476 ± 0.85	2.43 ± 0.83	0.343
Lipoprotein-a, mg/L	2.66 (2.18, 3.21)	2.60 (2.14, 3.10)	0.101
Hypersensitive troponin T, ng/L	224 (152, 340)	223 (180, 299)	0.587
N-terminal pro-brain natriuretic peptide, pg/ml	673.43 ± 49.58	676.30 ± 49.42	0.277
Lactate dehydrogenase, U/L	300.19 ± 159.92	293.92 ± 152.84	0.449
Killip classification, *n* (%)			0.644
1	193 (18%)	92 (18%)	
2	722 (66%)	347 (67%)	
3	182 (17%)	76 (15%)	
Calcified lesions, *n* (%)			0.725
Yes	562 (51%)	259 (50%)	
No	535 (49%)	256 (50%)	
Bifurcation lesions, *n* (%)			0.402
Yes	202 (18%)	86 (17%)	
No	895 (82%)	429 (83%)	
Ostial lesions, *n* (%)			0.185
Yes	117 (11%)	44 (9%)	
No	980 (89%)	471 (91%)	
Angular distortion lesions, *n* (%)			0.756
Yes	44 (4%)	19 (4%)	
No	1,053 (96%)	496 (96%)	
Target left main, *n* (%)			0.335
Yes	2 (0%)	3 (1%)	
No	1,095 (100%)	512 (99%)	
Target left anterior descending, *n* (%)			0.245
Yes	283 (26%)	147 (29%)	
No	814 (74%)	368 (71%)	
Target left circumflex, *n* (%)			0.822
Yes	394 (36%)	182 (35%)	
No	703 (64%)	333 (65%)	
Target right coronary artery, *n* (%)			0.11
Yes	352 (32%)	186 (36%)	
No	745 (68%)	329 (64%)	
Calcified lesions (NCL) *n* (%)			0.784
Yes	426 (39%)	196 (38%)	
No	671 (61%)	319 (62%)	
Bifurcation lesions (NCL), *n* (%)			0.739
Yes	224 (20%)	101 (20%)	
No	873 (80%)	414 (80%)	
Ostial lesions (NCL), *n* (%)			0.548
Yes	215 (20%)	108 (21%)	
No	882 (80%)	407 (79%)	
Angular distortion lesions (NCL), *n* (%)			0.948
Yes	232 (21%)	110 (21%)	
No	865 (79%)	405 (79%)	
NCL located on left anterior descending, *n* (%)			0.086
Yes	365 (33%)	149 (29%)	
No	732 (67%)	366 (71%)	
NCL located on left circumflex, *n* (%)			0.372
Yes	381 (35%)	191 (37%)	
No	716 (65%)	324 (63%)	
NCL located on right coronary artery, *n* (%)			0.361
Yes	347 (32%)	175 (34%)	
No	750 (68%)	340 (66%)	
Diameter stenosis, %	62.50 ± 6.98	61.98 ± 4.66	0.126
Lesion length, mm	13.77 ± 5.87	13.46 ± 5.84	0.324
Fractional flow reserve (FFR), *n* (%)			0.364
Yes	14 (1%)	9 (2%)	
NO	1,083 (99%)	506 (98%)	

ACEI, angiotensin converting enzyme inhibitor; ARB, angiotensin receptor inhibitor; NCL, non-culprit lesion.

**Table 2:** Univariate logistic regression analysis in the training group.

Variables	OR (95% CI)	*P*-value
Intervals between coronary angiograms, months	0.99 (0.98–1.01)	0.333
Age, years	1.09 (1.07–1.11)	**<0.001**
Sex, male vs. female	1.20 (0.83–1.72)	0.323
Diabetes mellitus	1.22 (0.83–1.76)	0.307
Hypertension	0.62 (0.44–0.86)	**0.005**
Smoking status	1.09 (0.78–1.52)	0.612
Alcohol drinking status	1.22 (0.75–1.91)	0.409
Coronary heart disease	0.99 (0.33–2.38)	0.98
Transient ischemic attack	2.83 (1.02–11.73)	0.084
Aspirin, *n* (%)	1.00 (1.00–1.00)	1
Clopidogrel, *n* (%)	0.97 (0.69–1.37)	0.856
Ticagrelor, *n* (%)	1.11 (0.78–1.56)	0.566
Beta-blockers, *n* (%)	1.10 (0.72–1.65)	0.637
ACEI/ARB, *n* (%)	0.86 (0.63–1.22)	0.434
Calcium channel blockers, *n* (%)	0.86 (0.63–1.22)	0.174
Statins, *n* (%)	2.21 (0.52–9.43)	0.283
High intensity of statin, *n* (%)	0.89 (0.63–1.27)	0.528
Door-to-balloon time (D-to-B)	1.00 (1.00–1.00)	0.552
Left ventricular ejection fraction, (%)	0.99 (0.97–1.00)	0.148
Creatine kinase MB, ng/ml	1.00 (1.00–1.01)	**0.001**
White blood cell, ×10^9^/L	0.97 (0.92–1.02)	0.282
Neutrophile, ×10^9^/L	1.02 (0.97–1.08)	0.45
Lymphocyte, ×10^9^/L	0.92 (0.75–1.12)	0.413
High-sensitive C-reactive protein (mg/L)	1.00 (0.99–1.01)	0.596
Fasting plasma glucose, mmol/L	0.94 (0.88–1.01)	0.083
Serum creatinine, umol/L	1.01 (1.00–1.01)	**<0.001**
Serum uric acid, umol/L	1.00 (1.00–1.00)	0.246
Total cholesterol, mmol/L	2.592 (2.18–3.08)	**<0.001**
Triglyceride, mmol/L	1.13 (0.99–1.27)	0.051
Triglycerides and glucose index	1.03 (1.01–1.05)	**0.005**
High density lipoprotein cholesterol, mmol/L	1.39 (0.90–2.10)	0.126
Low Density Lipoprotein cholesterol, mmol/L	0.88 (0.72–1.07)	0.202
Low Density Lipoprotein cholesterol on re-admission, mmol/L	1.06 (0.90–1.24)	0.488
Lipoprotein-a, mg/L	1.00 (1.00–1.00)	**<0.001**
Body mass index (kg/m^2^)	1.55 (1.44–1.67)	**<0.001**
Hypersensitive troponin T, ng/L	1.00 (1.00–1.00)	0.872
Lactate dehydrogenase, U/L	0.99 (0.98–1.00)	0.01
N-terminal pro-brain natriuretic peptide, pg/ml	0.99 (0.99–1.00)	0.091
Killip classification	1.76 (1.31–2.35)	**<0.001**
Calcified lesions	0.99 (0.71–1.38)	0.932
Bifurcation lesions	0.77 (0.48–1.20)	0.273
Ostial lesions	1.21 (0.70–1.98)	0.473
Angular distortion lesions	1.73 (0.80–3.45)	0.139
Target left main	0.91 (0.27–3.18)	0.997
Target left anterior descending	1.12 (0.76–1.61)	0.567
Target left circumflex	0.84 (0.58–1.19)	0.327
Target right coronary artery	0.71 (0.51–1.01)	0.052
Calcified lesions (NCL)	1.25 (0.88–1.78)	0.204
Bifurcation lesions (NCL)	1.47 (0.99–2.17)	0.054
Ostial lesions (NCL)	1.27 (0.86–1.89)	0.230
Angular distortion lesions (NCL)	0.71 (0.45–1.10)	0.122
NCL located on left anterior descending	0.78 (0.55–1.10)	0.156
NCL located on left circumflex	1.07 (0.76–1.52)	0.696
NCL located on right coronary artery	1.18 (0.82–1.69)	0.384
Diameter stenosis, %	0.98 (0.96–1.00)	0.122
Lesion length, mm	1.00 (0.97–1.03)	0.824
Fractional flow reserve (FFR), *n* (%)	1.73 (0.47–6.36)	0.685

ACEI, angiotensin converting enzyme inhibitor; ARB, angiotensin receptor inhibitor; NCL, non-culprit lesion.

Bold values indicate significant *P* < 0.05.

**Table 3:** Multivariate logistic regression analysis in training group.

Variable	OR (95% CI)	*P*-value
Serum creatinine, umol/L	1.007 (1.005–1.009)	**<0.001**
Age, years	1.091 (1.067–1.115)	**<0.001**
Body mass index (kg/m^2^)	1.476 (1.363–1.600)	**<0.001**
Triglyceride and glucose index	1.050 (1.022–1.079)	**<0.001**
Killip classification	1.594 (1.140–2.229)	**0.006**

Bold values indicate significant *P* < 0.05.

### Validation of the nomogram

**Figure 4:**
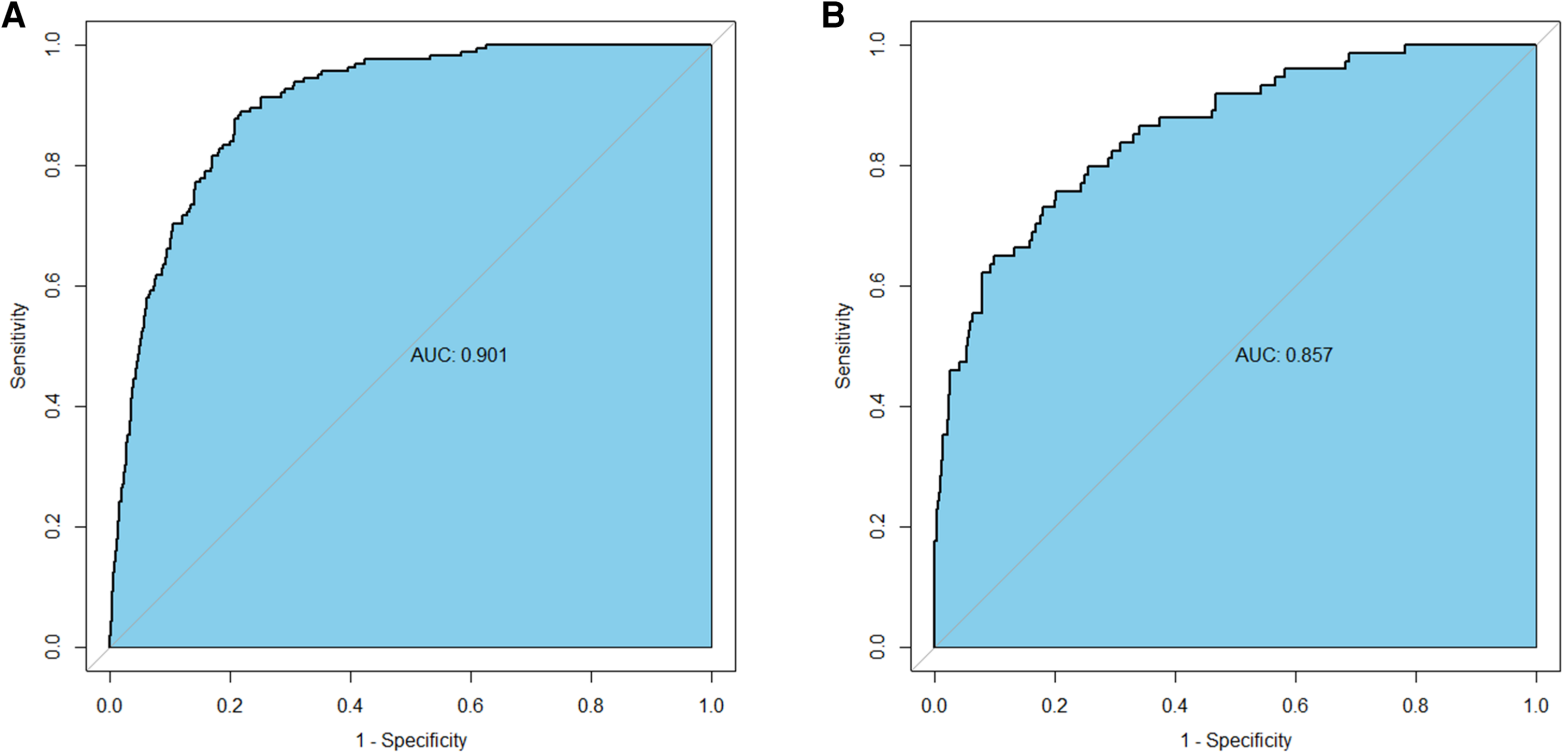
Receiver operating characteristics curve of the nomogram in the training group (**A**) and the validation group (**B**).

**Figure 5:**
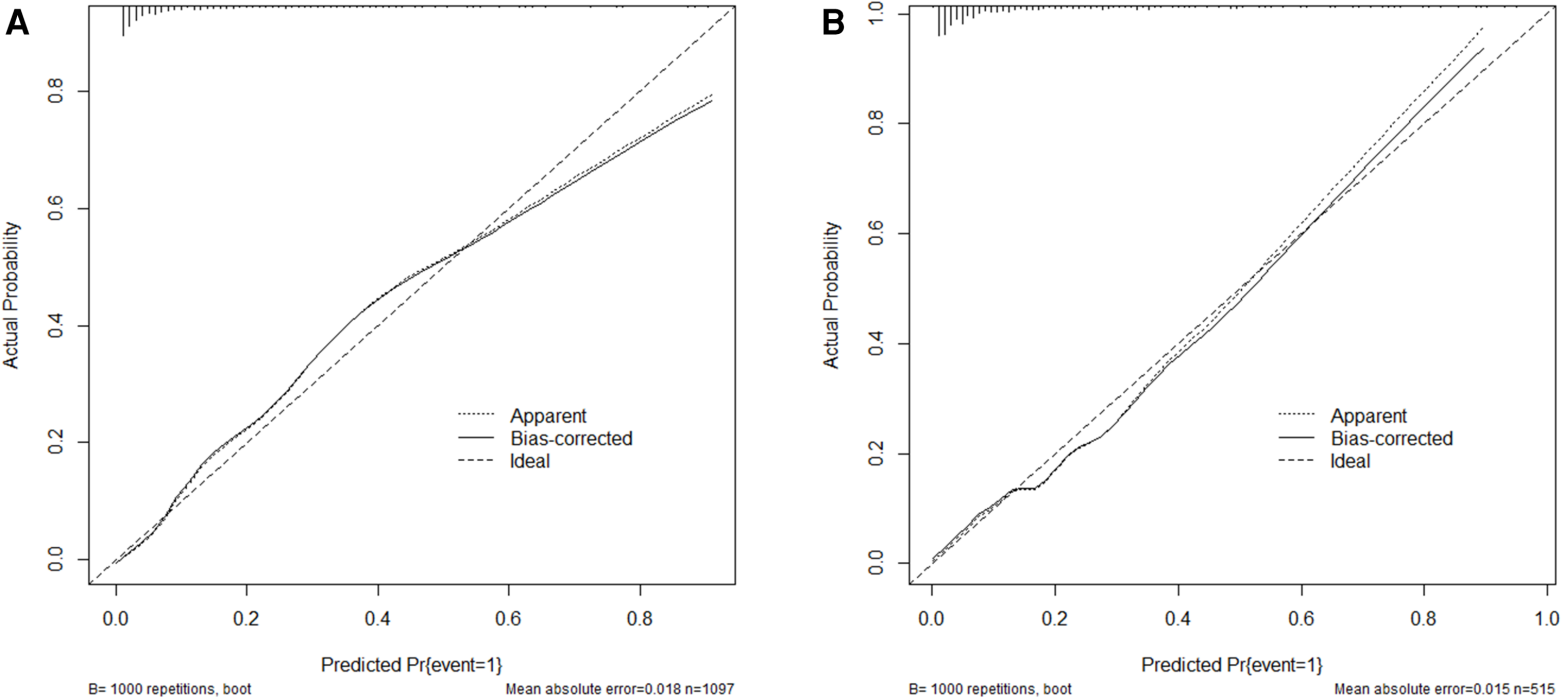
Calibration curve for the training group (**A**) and the validation group (**B**) the horizontal axis denotes the overall predicted probability of revascularization in STEMI patients after percutaneous coronary intervention due to non-culprit lesions, and the vertical axis displays the actual probability.

The AUC-ROC for the nomogram and validation sets (Figures 4A,B) was calculated to be 0.9014 (95% CI: 0.8795–0.9233) and 0.8574 (95% CI: 0.8103–0.9046), showing a good discrimination ability of the nomogram. The Hosmer-Lemeshow test produced *χ*^2^ values of the nomogram (7.3528, *P* = 0.4991) and the validation group (2.961, *P* = 0.9368), respectively. These results attest to the model's excellent calibration capability. Additionally, the calibration curve demonstrates high consistency between the predicted and actual probabilities (Figures 5A,B).

**Figure 6:**
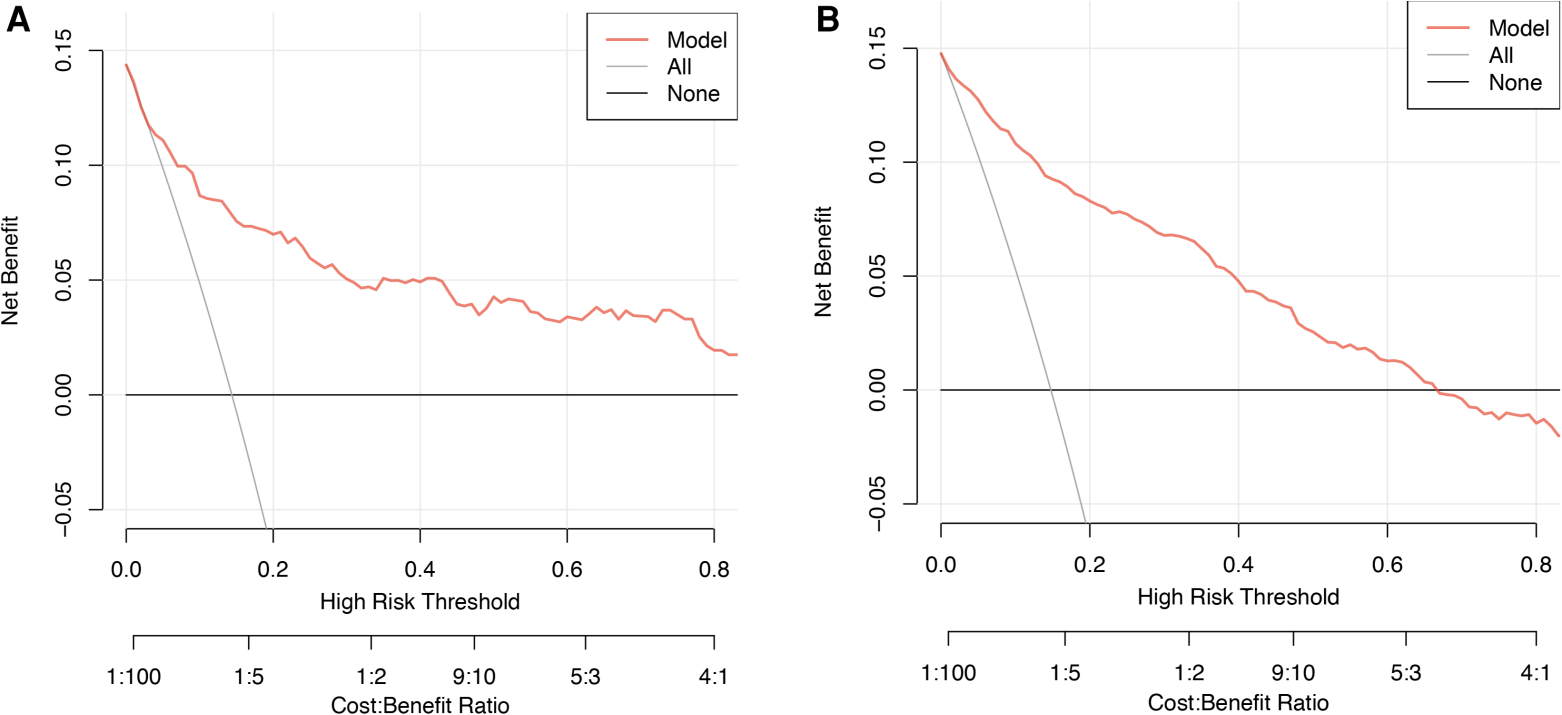
Decision curve analysis for the training group (**A**) and the validation group (**B**).

The clinical effectiveness of the model was evaluated, which is depicted in Figures 6A,B. The horizontal line indicates a net benefit of 0, which occurs when no intervention is performed and all samples are negative (Pi < Pt). Conversely, the green line corresponds to a scenario where all interventions are applied, and all samples are positive. The DCA demonstrates that the nomogram can achieve a net benefit over a wide range of threshold probabilities, indicating our approach's clinical utility.

## Discussion

With the aging of the population, there has been an annual increase in the incidence of acute ST-segment elevation myocardial infarction (STEMI). Fortunately, the prognosis for STEMI patients has improved with the widespread availability of percutaneous coronary intervention (PCI). Nevertheless, STEMI patients who have undergone PCI are still at risk of readmission due to in-stent restenosis or the non-culprit lesion (NCL) progression. The use of drug-eluting stents reduces incidences of in-stent restenosis as compared to bare metal stents. However, the application of drug-eluting stents has not demonstrated a commensurate improvement in the rate of revascularization. In two randomized controlled trials (16, 17), there was no difference between bare metal stent and drug-eluting stent applications for revascularization due to NCL progression in acute myocardial infarction (AMI) patients. Notably, numerous clinical trials (18–20) revealed that the rate of revascularization associated with NCL ranges from approximately 11.6%–19%, accounting for 42%–57% of all repeat procedures during the five years after PCI. It has been observed that while there have been several studies focusing on the progression of NCL, the repeated PCI for NCL progression has not received much attention. The study focuses on predicting repeat PCI due to NCL. One study of 480 patients identified fasting glucose and creatinine as independent risk factors for NCL progression (6), while another study of 492 patients suggested that several chronic stress and inflammation-related factors such as serum catecholamines, and C-reactive protein, as well as complex lesion rates, were related to the progression of NCL (7). We had a larger sample size (*n* = 1,612 vs. 480) and more complete clinical data (including angiographic, laboratory, and follow-up data) than previous studies, which increased the statistical power and generalizability of our results. Compared with traditional independent risk factors, nomograms could visualize the effect of predictors on NCL revascularization, which is a user-friendly tool to help clinicians and patients estimate the individualized risks and benefits of NCL PCI. Unlike previous studies that did not validate independent risk factors, we evaluated the validity of our model using ROC curve, calibration curve, and decision curve analyses both in the training and validation sets. The results showed that our model had good discrimination, calibration, and clinical utility. Therefore, the nomogram was useful and meaningful to predict NCL progression revascularization in STEMI patients who have undergone primary PCI.

To determine independent risk factors, a multivariate logistic regression analysis was performed. The variables analyzed included age, body mass index (BMI), triglycerides and glucose index (TyG), Killip classification, and serum creatinine (SCr). Furthermore, these five independent risk factors were integrated to build a nomogram to predict the revascularization for the progression of NCL in STEMI patients after PCI.

In the baseline characteristics, the population's age in the training and validation sets was 67 (57, 75) and 66 (56, 75) years. Notably, multivariate logistic regression results displayed that the likelihood of revascularization for NCL progression increased with advancing age. Based on statistical data, it is evident that the aging of the population has contributed to a steady rise in the occurrence of STEMI among older adults over the years. Currently, more than one-third of STEMI patients are over the age of 75 (21). In non-culprit lesions, plaque burden increases with age (22). Patients are mostly readmitted from acute coronary syndromes. In most acute coronary syndromes, thrombotic occlusion due to plaque rupture is considered a major pathological process (23). These plaques are generally characterized by a large plaque burden rich in lipid content. Unplanned revascularization is significantly more likely to occur in patients with large lipid plaques (24). The visceral and intramuscular fat ratio increases with age, reaching a peak between 60 and 75 years (25).

BMI is a valid indicator of obesity. According to the World Health Organization reported, worldwide obesity rates have almost tripled since 1975 (26). The risk ratios for repeat revascularization augment progressively with BMI increase, in other words, underweight patients have lower risk and severely obese patients possess higher risk (27). The mechanisms underlying the high risk of revascularization for NCL associated with high BMI are unclear. However, one potential explanation may be high BMI are associated with the impaired endothelium-dependent function of microvascular coronary arteries (28).

The TyG index is calculated from fasting triglycerides and blood glucose. A meta-analysis that included 5,731,29 patients showed that a higher TyG index may be independently associated with a higher incidence of atherosclerotic cardiovascular disease (29). TyG index is also a reliable alternative biomarker for assessing insulin resistance (30). Insulin resistance is a condition in which body tissues become resistant to insulin, leading to disturbances in lipid and glucose metabolism (31). These metabolic disturbances promote endothelial dysfunction, cardiovascular remodeling, oxidative stress, inflammatory factor release, and exacerbated blood pressure elevation, all of which may contribute to NCL revascularization after PCI (32, 33).

Killip classification is a simple clinical tool proposed by Killip et al. (34) in 1979 to quickly and effectively evaluate cardiac function. One study (35) found that Killip classification was associated with adverse cardiovascular events, including the progression of NCL. Another study (36) found that patients classified as a higher Killip classification tend to have more severe coronary artery injuries, which could eventually increase the likelihood of NCL progression. The coronary vascular endothelium in patients with a higher Killip classification is damaged by activation of the renin-angiotensin-aldosterone system and inflammatory storms (37, 38). These damages may be the cause of repeated PCI for NCL. Meanwhile, DeGeare et al. (39) reported that patients with high Killip classification were more susceptible to renal impairment after PCI. SCr can be a good indicator of kidney function. The prevalence of renal impairment increases with age. Severe renal impairment was reported that in 7% of patients aged 70%–80% and 11% of patients aged 80 years or older (40). Miyagi et al. (41) found a significant correlation between an increased percentage of lipid volume and decreased percentage of fibrous volume in NCL of patients with renal insufficiency. Furthermore, Hayano et al. (42) found that moderate chronic kidney disease (CKD) patients had more lipid and less fibrous volume in NCL. It has also been found that after standard lipid-lowering therapy after PCI in patients with CKD), lipid plaque regression occurred in CKD stage 1–2, while lipid plaque increase occurred in CKD stage 3–5. Fibrous plaques were also increased in CKD stage 4–5. People with CKD frequently present with obesity, hyperlipidemia, hyperglycemia, and hypertension, all of which are risk factors that make them susceptible to atherosclerosis development. At the same time, chronic inflammation and stress can lead to maladaptive repair responses that destabilize plaque. Unstable coronary atheromatous plaque rupture often leads to downstream coronary artery obstruction (43). Besides, SCr concentrations are thought to be associated with oxidative stress, endothelial dysfunction, and more progressive atherosclerosis (44, 45). These biological abnormalities may also contribute to the risk of NCL revascularization.

Progression of NCL is responsible for more than half of the causes of revascularization after PCI in STEMI patients, but not all NCLs require revascularization. In this regard, identifying high-risk groups is critical. The nomogram in the study was constructed using 5 variables that were easy to obtain at the time of patient admission. The model can accurately and reliably assess the risk of NCL revascularization in STEMI patients after PCI. In clinical practice, clinicians can use the nomogram can be used to quantify the weighting of risk factors to obtain a total score based on the patient's condition. The total score could be used to locate the corresponding 5-year probability of NCL revascularization on the nomogram scale. Further, clinicians should use the probability of NCL revascularization to guide the management of NCL in patients with STEMI. For example, if the probability is high (>50%), clinicians may choose to intensify medical treatment and follow-up of NCL to avoid possible intervention. If the probability is very low (<10%), it can reduce the patient's concern and psychological burden. If the probability is intermediate (10%–50%), clinicians can discuss the importance of medical therapy follow-up with patients, and make a joint decision based on the patient's preferences and clinical situation. Therefore, the tool is useful for clinicians to identify and target high-risk groups as early as possible.

### Limitations

Limitations of the study should be acknowledged. Firstly, this is a retrospective study. Not all STEMI patients who received PCI at our hospital were included in the study, as some patients may choose to seek consultation elsewhere when NCL progression occurred again. This situation may have resulted in some bias in the study's findings. Secondly, although the study incorporated common clinical indicators, there were certain factors that were not taken into consideration. For instance, the research did not account for serum catecholamines, a risk factor for NCL progression. This omission may have implications for the interpretation of the study's findings. Thirdly, our study involved a retrospective analysis of readmitted patients who underwent NCL PCI. Therefore, our focus was on NCL revascularization as the primary endpoint, which is a common and clinically relevant outcome in patients with STEMI. Other endpoints, such as death, acute myocardial infarction, and culprit revascularization, were also important and could potentially impact the outcome of NCL PCI. However, we did not include them in this study. In terms of adjudicating endpoint events, we followed the definitions and methods documented in the hospital records. We acknowledge that this reliance on retrospective data may introduce inherent bias, a limitation inherent in this type of study. Fourthly, even when NCL progression occurs, patients do not tend to seek immediate medical care. Therefore, the time of readmission is not necessarily the time of NCL progression. We performed logistic regression rather than Cox regression for the detection of predictors of 5-year NCL-related revascularization because we were interested in the binary outcome of whether or not the revascularization occurred within 5 years, not in the time to revascularization. However, we also recognize that time to revascularization and revascularization should be considered together, but as a retrospective study, the absence of a rigorous follow-up mechanism is a major limitation. Finally, although the study had external validation, the data analyzed were collected from different periods within a single hospital. The nomogram's clinical value should be evaluated further using multicenter and larger sample sizes in future studies.

## Conclusion

A convenient and accurate nomogram was developed and validated using five factors for predicting the revascularization due to NCL progression in STEMI patients after primary PCI. The nomogram enables clinicians to make appropriate disease management decisions by assessing the risk of NCL progression revascularization for STEMI patients after primary PCI.

## Data Availability

The raw data supporting the conclusions of this article will be made available by the authors, without undue reservation.
